# The Role of Pharmacies in Providing Point-of-Care Services in the Era of Digital Health and Artificial Intelligence: An Updated Review of Technologies, Regulation and Socioeconomic Considerations

**DOI:** 10.3390/healthcare14030309

**Published:** 2026-01-26

**Authors:** Maria Daoutakou, Spyridon Kintzios

**Affiliations:** 1 Laboratory of Cell Technology, Department of Biotechnology, Agricultural University of Athens, 11855 Athens, Greece; daoutakou@aua.gr; 2 Key2Core, 11141 Athens, Greece

**Keywords:** artificial intelligence, community pharmacy, biosensor, diagnostics, digital health, pharmacy, point-of-care testing, quality control, regulation, telepharmacy

## Abstract

Pharmacy-based point-of-care (POC) services have evolved from pilot initiatives to an essential component of decentralized healthcare delivery. These services—ranging from rapid infectious-disease screening to chronic-disease monitoring—improve access, reduce diagnostic delays and empower pharmacists as front-line healthcare providers. The present paper is an updated, in-depth review of the evolution of pharmacy POC services worldwide, combined with the analysis of the regulatory and educational frameworks supporting implementation, technological drivers such as biosensors, mobile health and artificial intelligence and in-depth socioeconomic considerations. Benefits for patients, pharmacies and healthcare systems are contrasted with challenges including variable reimbursement, uneven regulatory oversight and workforce preparedness.

## 1. Introduction to Point-of-Care Services in Pharmacies

Community pharmacies are becoming vital nodes in the emerging analytical health network that connects diagnostic chemistry with decentralized clinical practice. Traditionally restricted to dispensing and counseling, pharmacies are now transitioning into localized analytical laboratories where rapid chemical and biochemical measurements are performed at or near the site of patient care. This transition is enabled by the miniaturization of analytical instrumentation, the development of robust biosensing chemistries and the evolution of regulatory frameworks that recognize pharmacists as qualified operators of analytical devices [1,2].

**Figure 1:**
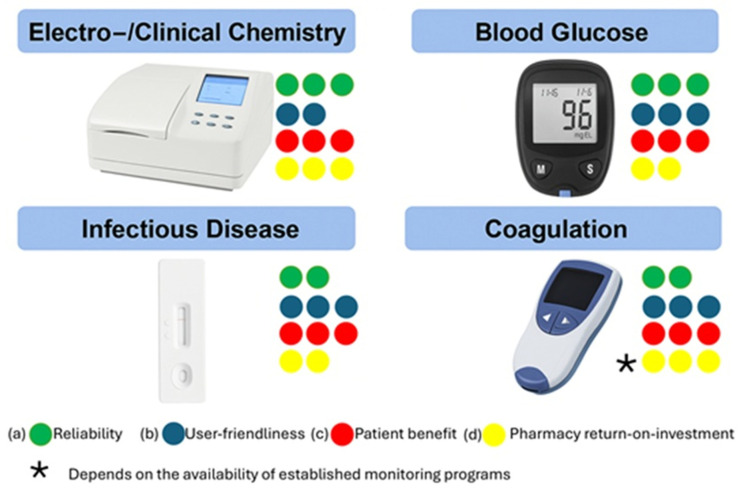
Key examples of analytical and biosensing POCTs applied in pharmacy sensing. The four different color codes and respective rankings (1–3) regard the (a) reliability, (b) user-friendliness (training needed for pharmacists), (c) patient benefit (regarding disease management) and (d) pharmacy-based return on investment (economic impact for pharmacies) for each POCT category.

POC testing (POCT), in its strict analytical sense, refers to the in situ measurement of clinically relevant analytes in biological matrices such as blood, saliva or urine using portable or benchtop devices capable of producing quantitative or semi-quantitative results within minutes. The underlying analytical systems are frequently based on electrochemical, optical or immunochemical detection schemes that convert a biochemical recognition event into a measurable signal (Figure 1). Typical analytes encountered in community pharmacy practice include glucose, glycated hemoglobin (HbA1c), C-reactive protein (CRP), cholesterol, triglycerides, troponin I/T and markers of infectious diseases such as influenza or SARS-CoV-2 antigens [3,4].

The COVID-19 pandemic underscored the strategic relevance of distributed analytical capacity. During global lockdowns, pharmacies maintained open access and rapidly deployed antigen and antibody lateral-flow immunoassays under emergency authorization. Subsequent studies demonstrated that pharmacy-performed tests could achieve analytical sensitivities above 90% and specificities exceeding 95% when manufacturer protocols and external quality controls were applied [5,6,7]. This experience catalyzed a paradigm shift: pharmacies are no longer viewed merely as dispensing outlets but as chemically competent micro-laboratories within the healthcare ecosystem [8].

The analytical rationale for integrating pharmacies into POC testing lies in both proximity and chemical fidelity. By reducing the pre-analytical delay—the interval between sample collection and analysis—the integrity of labile analytes (e.g., glucose, lactate, ammonia and certain enzymes) is preserved [9]. Consequently, analytical errors attributable to sample degradation or mishandling decrease dramatically compared with centralized laboratory workflows.

From an analytical chemistry perspective, the expansion of POC testing to pharmacies introduces a new class of end users—trained but non-specialist analytical operators—into the diagnostic value chain. This requires instrument designs that are chemically stable, reagent-integrated and largely automated in calibration and quality control [10]. Cartridge-based reagent systems employ lyophilized or micro-encapsulated chemistries that can withstand variable storage conditions and provide reproducible rehydration kinetics at the moment of testing. The analytical accuracy of such devices is sustained through built-in electrochemical reference systems, optical calibration curves stored on embedded chips and algorithmic temperature compensation [11].

**Figure 2:**
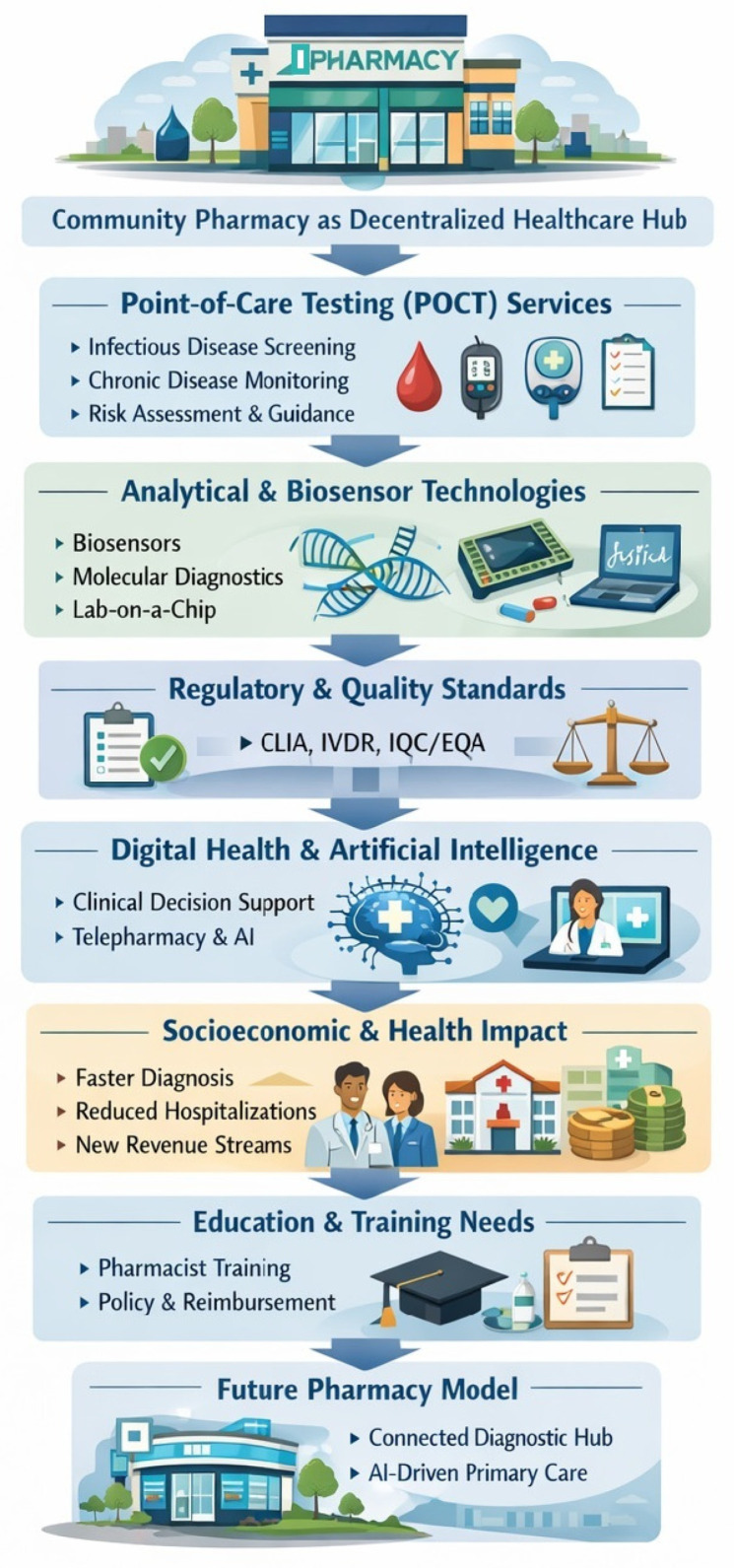
Graphical summary of the structure of the present review of pharmacy-based POCT services.

Previous reviews [1,12,13,14] have focused on the increasing role of pharmacies in providing POCT services for communicable and non-communicable diseases, especially in low- and middle-income countries. However, the advent of both the COVID-19 pandemic and AI-based applications in digital health has reshaped the on-site testing, pharmacy-based landscape. Therefore, the current review examines the chemical and analytical foundations that enable point-of-care testing in pharmacies, the regulatory infrastructures that guarantee quality and safety and the technological architecture ranging from electrochemical transducers to AI-driven data analytics—that define next-generation diagnostic practice, along with challenges related to economic sustainability of POCT services and the pharmacists’ educational framework (Figure 2).

## 2. Evolution and Regulatory Context of Pharmacy-Based Point-of-Care Services

### 2.1. The Regulatory Landscape

Pharmacies have been a societal mainstay through the delivery of prescription drugs and, to a lesser extent, other products. There is, however, confusion among the public and even the health care community about the definition of and services available from pharmacies, which is a contributing factor to the slow acceptance of pharmacies as full-fledged providers of comprehensive health care [15,16].

The successful implementation of point-of-care services by pharmacies is predicated upon the existence of an appropriate regulatory framework that clearly delineates the scope of practice for pharmacies. The regulatory recognition of pharmacies as analytical testing environments has progressed unevenly across jurisdictions. Early legislative frameworks in Europe and North America focused primarily on *good dispensing practice* and the stability of pharmaceutical formulations rather than on analytical measurement performed on biological matrices [17]. The introduction of glucose self-testing in the 1980s marked the first major interface between clinical chemistry and community retail environments [18]. Subsequent regulatory milestones—such as the U.S. Clinical Laboratory Improvement Amendments (CLIA, 1988) and the European IVD Directive 98/79/EC (superseded by the IVDR 2017/746)—created the legal category of “waived” or “near-patient” tests [19,20,21]. The CLIA waiver is a certification granted by the Centers for Medicare & Medicaid Services (CMS) in the United States. It allows laboratories, including community pharmacies, to perform specific diagnostic tests that are categorized as “waived” [22]. In Europe, the IVD Directive 98/79/EC established the requirements for marketing in vitro diagnostic medical devices (IVDs) in the EU, allowing most devices to be self-certified with a CE mark. It has since been superseded by the more rigorous In Vitro Diagnostic Regulation (IVDR) 2017/746, which became fully applicable on 26 May 2022, to provide greater patient safety and address significant technological advancements in the field [23]. The IVDR introduces a risk-based classification system and requires more stringent conformity assessments, with the majority of IVDs now needing a notified body’s involvement for certification. These tests are simple to perform, have a low risk of erroneous results and do not require specialized laboratory personnel or complex equipment. These categories permit operation by non-laboratory professionals provided that analytical systems meet predefined performance and risk criteria. In particular, CLIA-waived POCTs available for use in community pharmacies include (a) antibody/antigen-based lateral flow immunoassays and (b) molecular-based tests employing nucleic acid amplification technologies (NAATs), both for a variety of targets (Table 1).

Lateral flow assays offer the advantage of rapid results (usually 5–30 min), low cost and ease of use, although visual result interpretation may be subjective, and the employment of a reader may be necessary for certain tests. Molecular methods, on the other hand, are highly sensitive and specific, offer multiplex capabilities and are considered more reliable than immunological methods. On the cons side, they are generally characterized by higher cost, longer run times, specific storage requirements for reagents and limited throughput capacity.

From a chemical-analytical standpoint, the challenge for regulators is to ensure that simplified devices still adhere to fundamental principles of metrology—traceability, accuracy, precision, linearity and detection limit—while being operable in non-controlled environments. For pharmacies, this translates into the need for standardized protocols addressing

1. *Analytical validation*—verification of calibration linearity, limit of detection (LOD), limit of quantification (LOQ) and coefficient of variation (CV%) against reference laboratory methods [37].
2. *External quality assessment (EQA)*—participation in proficiency-testing schemes organized by accredited laboratories [38].
3. *Internal quality control (IQC)*—routine checks using control materials of known concentration; statistical monitoring via Levey–Jennings plots [39].
4. *Documentation and traceability*—use of barcode-encoded reagents and electronic result logging compliant with data-protection legislation [40].

The analytical reliability of pharmacy-based testing also depends on environmental control. Unlike centralized laboratories, pharmacies may experience fluctuating temperature and humidity, especially in resource-limiting settings [41]; thus, devices should ideally incorporate solid-state temperature sensors and correction algorithms. Reagent cartridges should be sealed in foil pouches with desiccants, and on-board calibration curves should be periodically updated through software revisions approved by notified bodies [42].

Regulatory evolution increasingly recognizes the *chemometric* dimension of diagnostics—where measurement uncertainty is not only instrumental but also algorithmic. Under the new European IVDR, manufacturers must provide performance evaluation reports including statistical parameters such as slope, intercept, R^2^ and bias versus reference methods, explicitly linking these to clinical-decision limits. Pharmacies implementing such systems must maintain a *Quality Management System* (QMS) comparable to ISO standards, adapted to community settings.

Beyond the USA and Europe, regulations regarding POCT in pharmacies may vary. In Australia, for example, the Therapeutic Goods Administration (TGA) classifies pharmacy POC tests as Class 2 in vitro diagnostic devices requiring analytical performance data on sensitivity, specificity, repeatability and stability [43]. Canada’s Health Canada and the U.S. FDA employ similar frameworks but emphasize *risk-based* categorization (e.g., CLIA-waived, moderate or high complexity). Pharmacies usually operate within the CLIA-waived category, meaning the analytical method must be simple, with a negligible risk of erroneous results [44].

The implication for analytical chemists is profound: regulatory compliance becomes an extension of analytical validation. Instrument calibration, reagent formulation and chemometric algorithms are all audited for reproducibility under real-world use. This ensures that the chemical measurement principles—electrode potential, absorbance, luminescence or antigen–antibody binding kinetics—remain quantitatively traceable to national metrology standards even outside traditional laboratories.

### 2.2. Internal and External Quality Control

Quality control (QC) procedures in pharmacies mirror those in laboratories, adapted to smaller sample throughput. Two control levels (low/high) should be tested at least daily. Results are plotted on Levey–Jennings charts, enabling visual detection of systematic bias [45]. Westgard multirule criteria (1 2s, 1 3s, 2 2s, R 4s, 4 1s, 10×) guide accept/reject decisions [46].

That said, participation in external quality assessment (EQA) programs remains the hallmark of analytical credibility. All participating sites (labs or pharmacies) measure the same blinded control sample. Their results form a *peer group distribution*. The peer group mean and peer group standard deviation (SDpeer) describe how the group performed collectively.

EQA samples are blinded materials analyzed quarterly; *z-scores* are computed as

z=(xi−xmean)/SDpeer

where |*z*| ≤ 2 indicates satisfactory performance [47]. WHO, IFCC and ISO jointly coordinate global EQA schemes. Pharmacies participating through national hubs send monthly QC results to central databases, where *z-scores* are computed and corrective actions recommended [48].

Global interoperability requires machine-readable analytical data using standardized metadata (analyte ID, method, device lot, calibration file checksum). HL7 FHIR and LOINC codes are now extended to POC devices; for example, LOINC 1558-6 denotes *Glucose BldC POC* [49]. Chemometric compression algorithms (e.g., Principal Component Encoding) allow efficient cloud transfer of high-frequency amperometric data while preserving signal-to-noise ratios.

### 2.3. Regional Implementation Models

#### 2.3.1. Europe

In the EU, the *Joint Committee for Traceability in Laboratory Medicine (JCTLM)* lists reference methods for creatinine (IDMS), cholesterol (GC-MS) and HbA1c (HPLC) [50]. POC device manufacturers claiming equivalence must demonstrate analytical agreement (slope 0.95–1.05; intercept ±0.1 units; R^2^ ≥ 0.97) versus these reference methods under ISO 17511.

European pharmacies integrate diagnostic chemistry within publicly funded health systems. The UK’s *Community Pharmacy Consultation Service* authorizes CRP, lipid and influenza POC testing under NHS accreditation [51]. Devices must comply with CE-marking under IVDR 2017/746, which now requires analytical-performance studies, risk analysis (ISO 14971) and clinical-evidence dossiers. Germany’s *Apothekenlabor* model emphasizes continuous external QC participation (Richtlinie BÄK 2021) [52]

#### 2.3.2. North America

U.S. pharmacies operate under CLIA-waived classification. Analytical chemistry aspects include simplified calibration protocols and on-board controls validated against CLSI EP15 guidelines (precision evaluation using ≤5 replicates × 5 days) [53]. Canada’s provincial authorities (e.g., New Brunswick College of Pharmacists) require adherence to *Good Laboratory Practice for Point-of-Care Testing*, including traceability to IDMS methods for glucose and creatinine [54].

#### 2.3.3. Asia-Pacific

Australia’s *GuildCare POCT* program mandates analytical validation prior to public reimbursement [55]. Japanese pharmacies integrate micro-spectrophotometric analyzers under *Yakkyoku Shindan Senta* (Pharmacy Diagnosis Centers), maintaining calibration certificates issued by the Japan Quality Assurance Organization (JQA) [56].

### 2.4. Risk Management and Chemical Safety

Pharmacies handling biological samples must adhere to biosafety levels 1–2. Chemical risk analysis includes proper disposal of reagents containing peroxides, azides or chromogenic substrates (e.g., TMB, ABTS) [57]. Buffer solutions are neutralized before disposal; micro-quantities of heavy-metal salts from electrochemical reference systems are collected as hazardous waste. Integration of *micro-total-analysis systems (µTAS)* reduces chemical waste to microliter volumes, aligning with green-chemistry principles.

## 3. Biosensor Technologies and Diagnostic Integration

**Figure 3:**
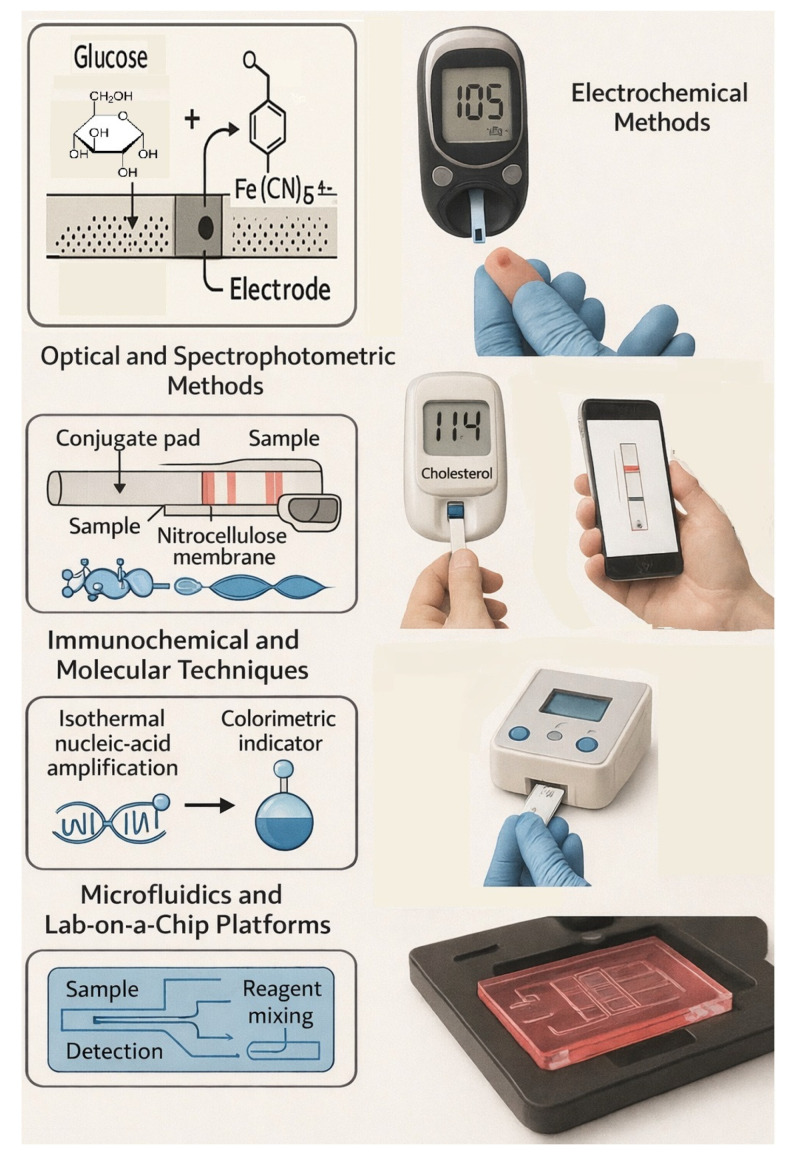
Overview of different biosensor technologies used in pharmacy-based POCT.

The technological basis of POC testing in pharmacies encompasses a broad spectrum of analytical, biosensor-based transduction mechanisms unified by the goal of *rapid, accurate and miniaturized quantification*. At the heart of each device is a chemical or biochemical recognition element coupled to a physical transducer that converts a molecular event into an electrical or optical signal (Figure 3).

### 3.1. Electrochemical Systems

Electrochemical biosensors dominate pharmacy-grade diagnostics because they combine chemical specificity with low cost and straightforward miniaturization. Amperometric glucose meters—based on glucose oxidase or glucose dehydrogenase catalysis—remain the canonical example. The enzymatic oxidation of glucose generates electrons transferred via mediators (ferricyanide, ferrocene derivatives) to screen-printed carbon electrodes. Modern devices incorporate nanostructured materials (graphene, carbon nanotubes, Au NPs) to enhance electron-transfer kinetics and lower detection limits [58].

Beyond glucose, amperometric or potentiometric principles are applied to cholesterol, lactate, uric acid and electrolytes. Disposable strip-based sensors utilize thin-film microfabrication and integrated reference electrodes (Ag/AgCl) for stability. Analytical performance routinely reaches CV < 5% with correlation coefficients > 0.92 versus standard clinical analyzers [59], including glucose, lactose, Hb, uric acid, and cholesterol testing and total blood cell count.

**Figure 4:**
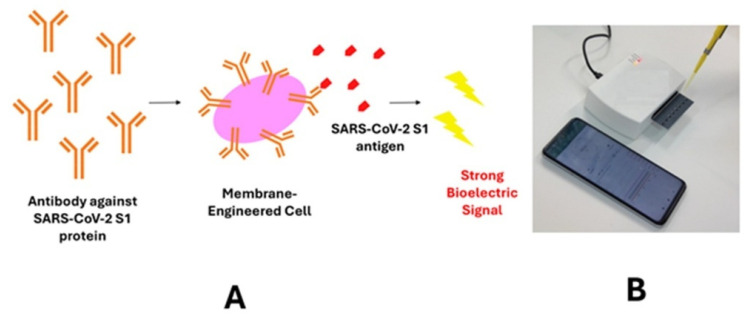
Presentation of (**A**) the working concept of the membrane-engineered, cell-based bioelectric biosensor for screening for SARS-CoV-2 and (**B**) the actual test device with smartphone display.

An even more advanced version of POC electrochemical biosensors is represented by the cell-based bioelectric biosensor developed by Mavrikou et al. [60] in which living mammalian cells act as the primary biorecognition and signal-transduction elements. The application of this novel technology was demonstrated in the clinical screening for SARS-CoV-2 in saliva using membrane-engineered SK-N-SH cells bearing electroinserted human anti-SARS-CoV-2 S1 antibodies, which transduced antigen–antibody binding events into rapid, measurable changes in cell membrane potential, recorded via a portable potentiometric platform (Figure 4).

### 3.2. Optical and Spectrophotometric Methods

Optical POC systems convert chemical concentration into absorbance, fluorescence or luminescence signals. Photometric lateral-flow assays for infectious diseases use colloidal-gold or latex beads producing color changes detectable by smartphone cameras [61]. Quantitative reflectance photometry—employed in cholesterol and HbA1c meters—relies on LED sources and photodiode detectors calibrated for pathlength and wavelength drift. Chemiluminescent immunoassays (CLIA) are emerging in compact benchtop pharmacy analyzers, providing analytical sensitivity down to pg mL^−1^ for hormonal or viral markers [62].

### 3.3. Immunochemical and Molecular Techniques

Pharmacies increasingly deploy immunochemical POC tests utilizing antibody–antigen specificity. The analytical chemistry of these systems depends on surface functionalization of nitrocellulose or polymer membranes with capture antibodies and on enzyme-labeled or nanoparticle-conjugated detection antibodies. Signal intensity correlates with analyte concentration through enzyme kinetics or nanoparticle optical density.

Molecular POC diagnostics—e.g., isothermal nucleic-acid amplification (LAMP, RPA)—are entering pharmacy settings for infectious-disease screening. These methods rely on strand-displacing polymerases and colorimetric indicators (hydroxynaphthol blue, SYBR Green) to report amplification products without thermal cycling. Analytical times of 15–30 min with LODs below 10^2^ copies μL^−1^ are typical [63]. Cartridge-integrated microheaters ensure precise temperature control critical for reaction fidelity.

### 3.4. Microfluidics and Lab-on-a-Chip Platforms

Microfluidic systems miniaturize entire analytical workflows—sample metering, reagent mixing, reaction, separation and detection—onto polymer chips fabricated via soft lithography. These devices handle microliter volumes, reducing reagent consumption and improving reaction kinetics through enhanced surface-to-volume ratios [64]. For pharmacy use, disposable microchips preloaded with reagents enable multiplex analysis of lipids, glucose and inflammatory markers. Capillary forces or centrifugal motion drive fluid transport; detection is performed electrochemically or optically [65].

Chemical compatibility and stability are key challenges: reagents must remain active over shelf lives exceeding one year under ambient storage. Encapsulation techniques (sol–gel matrices, sugar-based stabilizers) preserve enzymatic or antibody activity, while on-chip drying under controlled humidity ensures reproducible reconstitution upon sample addition [66].

### 3.5. Integration, Data Handling and Chemometrics

Modern pharmacy analyzers integrate sensors with microcontrollers capable of performing calibration, baseline correction and chemometric modeling. Algorithms based on partial-least-squares (PLS) regression, principal component analysis (PCA) or neural-network classifiers interpret multivariate signals, distinguishing true analyte responses from background noise [67]. Such processing transforms raw amperometric or optical data into clinically actionable concentrations.

The chemical performance of POC devices is evaluated using key analytical metrics such as

**-**  Sensitivity (S) = Δ_Signal_/Δ_Concentration_
**-**  Limit of detection (LOD) = 3σ/S
**-**  Selectivity determined by cross-reactivity studies with structurally related analytes
**-**  Repeatability and reproducibility expressed as intra- and inter-assay CVs
**-**  Analytical recovery using spiked control materials.

Reported LODs for modern electrochemical POC sensors reach 10^−6^–10^−9^ M for metabolites and 10^−12^ M for immunoassays, bridging the gap between benchtop analyzers and laboratory instruments [68].

Integration with telehealth systems allows pharmacists to transmit chemically validated results to physicians within minutes, closing the analytical loop between measurement and medical decision. Connectivity is achieved through Wi-Fi or Bluetooth modules, allowing automatic upload of anonymized data to cloud servers for epidemiological aggregation. Data integrity is ensured via checksum verification and encryption compliant with the General Data Protection Regulation (GDPR) and Health Insurance Portability and Accountability Act (HIPAA). Pharmacies thus become *chemical data nodes*—autonomous yet networked laboratories contributing real-time analytical information to population-level health monitoring.

## 4. Benefits, Challenges and Economic Considerations of Point-of-Care Services in Pharmacies

### 4.1. Benefits and Challenges

An increasing number of pharmacies around the world are providing POC services, which include a variety of clinical services such as screening, diagnostic tests, health risk assessment and monitoring of disease or medication. This process is being partly driven by the need for increased access, chronic disease management, and the legacy of the COVID-19 pandemic, but its dynamic differs among regions. Roughly 51.6% of U.S. community pharmacies hold a CLIA-waiver, a massive increase from 17.9% in 2015. In some states, adoption is as high as 87.9% (https://seed.nih.gov/sites/default/files/2024-12/CLIA-Waived-Tests.pdf (accessed on 3 January 2026). Practice in Europe is fragmented due to country-specific regulations, though the European POCT market is projected to reach over USD 33.6 billion by 2033 with a growth rate of 9.4% [69].

Several studies have suggested benefits of POC services related to the healthcare system, pharmacy sector, patients and society overall; yet, in contrast, numerous challenges have also been reported. For example, in their 2020 systematic review of community pharmacy POCT, Albarsi et al. [13] identified 13 studies (covering over 23,000 patients, though many were in primary care settings, with a growing number specific to pharmacies). A preliminary pilot study on CRP-POCT in community pharmacies showed a 16% reduction in non-prescription antibiotic dispensing [70]. Using the same example, Martínez-González et al. [71] reviewed 13 studies comprising 9844 participants for evidence on the impact of CRP-POCT on antibiotic prescribing for respiratory tract infections in primary care. They found that POC tests significantly reduced immediate antibiotic prescribing compared with usual care (RR 0.79, 95% CI 0.70 to 0.90). In the case of glycemic control, as another example, Al Hayek et al. [72] reported the results of a study including 75 diabetic patients where the mean HbA1c level significantly improved after POCT implementation compared to the traditional HbA1c laboratory testing before POCT implementation (8.34  ±  0.67 and 8.06  ±  0.62, respectively, *p*  <  0.001).

**Figure 5:**
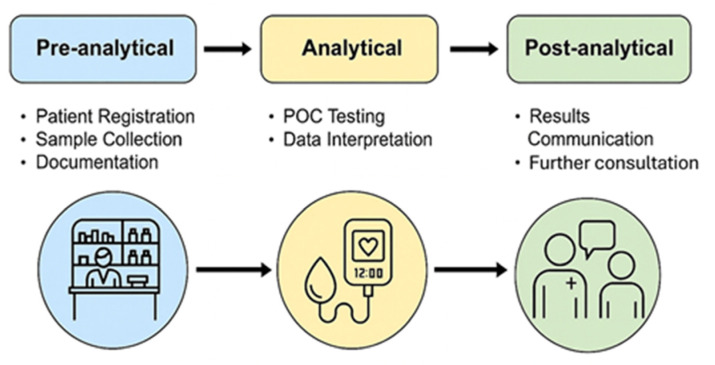
Workflow of pharmacy-based POCT service delivery.

On a broader level, a healthcare system benefit involving POC services is the decrease in morbidity and mortality and better health management among the population. POC services in pharmacies have the potential to improve outcomes for patients with chronic illnesses, thereby reducing hospital admissions and the overall burden on health services. An illustrative example is glycemic control, mentioned above, as well as early interventions in the diagnosis and treatment of respiratory tract infections, evidently experienced during the COVID-19 pandemic and the wide application of rapid tests at pharmacies. Another benefit could be the impact on reducing the spread of communicable disease, e.g., by increasing HIV POCT (see also Section 7 below). With respect to pharmacy sector benefits, the competitive edge and profitability of pharmacies can be improved as a result of different revenue streams generated from POC services. Moreover, pharmacies providing professional POC services are likely to attract more foot traffic, increasing the sale of both medications and over-the-counter products [72]. From the perspective of patients, closer monitoring helps improve the effectiveness of drug therapy. Promptly receiving the test results and medical advice can benefit the patients’ health status, particularly in emergency cases. The societal benefit stated is drug resistance prevention, currently an important global concern. POC tests help adhere to good prescribing practices, hence improving the quality and safety of treatment [73] (Figure 5).

Challenges of POC services in pharmacies are also numerous. One major issue is the unsound knowledge of existing POC technologies, devices, tests and services, which appears to be common among pharmacists as well as pharmacy owners. Other challenges include the lack of necessary facilities at pharmacies to set up POC services, inappropriate reimbursement policies in the case of unpaid professional service, concerns regarding false results from devices employed, poor perception of test inaccuracy among pharmacy staff and a shortage of trained staff to carry out the POC tests [74,75]. Lack of managerial vision as to how the pharmacy will be fitted into the delivery of healthcare services in terms of POC or non-POC testing was also cited as a barrier to the implementation of POC services with reference to the pharmacy owner [14,76]. Such barriers pointed at the pharmacy sector are likely to discourage pharmacists from using or implementing POC services. Despite the challenges mentioned above, pharmacy-based POC services present numerous advantages and great prospects in countries where pharmacies are easily reachable. As part of the larger healthcare team, pharmacists can contribute positively to improving health outcomes. To promote the sustainable development of pharmacy-based POC services, opportunities to increase awareness and understanding of POC services must be created among pharmacy staff.

Benefits and challenges of pharmacy-based POC services are summarized in the following Table 2:

### 4.2. Economic, Policy and Sustainability Considerations

The economics of POC diagnostics depend on the *cost-per-test (CPT)* and the *cost-per-accurate-result (CPAR)*, where

*CPAR* = *CPT*/(1 − *p_error*)

If analytical error probability (*p_error*) = 0.05, a EUR 5 test effectively costs EUR 5.26 per reliable measurement. Thus, investments in calibration and QC reduce overall healthcare expenditure by lowering *p_error* [77].

In order to adopt a policy of providing POCT as a service, capital costs per pharmacy would include equipment costs (for molecular, automated and/or high-throughput analysis), quality testing reagents, test reagents and associated software [78]. The estimated cost for said investment is broken down in the following Table 3:

For pharmacies offering POCT services, economic models show break-even at ~600 tests/year when reimbursement ≥EUR 7/test [79,80,81]. In addition, a cost–benefit meta-model shows return on investment > 300% within 3 years for networks exceeding 100 pharmacies [82]. On the other hand, reducing diagnostic (analytical) uncertainty doubles the so-called *Value-of-Information (VoI)* index, as described by the following equation:

*VoI* = *ΔHealth Outcome*/*Analytical Uncertainty*

In other words, reducing analytical uncertainty from ±10 to ±5% will double *VoI*, even if equipment costs rise by 20%. Hence, public health funding agencies and insurance companies increasingly evaluate diagnostics through Value-of-Information rather than unit cost [83]. In the same context, chemometric analyses of national datasets report a cost elasticity with respect to analytical precision in the order of −EUR 0.8 million per 1% precision gain. Thus, for every 1% improvement in CV, a reduction of national diagnostic costs on the order of ca. EUR 800,000/year could be expected for mid-size countries and economies [84]. Moreover, quantitative risk assessment models express *Net Analytical Benefit (NAB)* according to the following equation:

*NAB* = (*Clinical Benefit* × *Accuracy*) − (*Cost × Error Probability*)

whereas sensitivity analysis reveals a positive NAB at accuracy levels higher than 92%, even at test cost levels of EUR 10 [85]. In other words, improving diagnostic accuracy yields exponential societal and economic returns.

In the context of public health policy, a model proposed by the OECD in 2024 showed that adoption of a nationwide pharmacy-based POC testing framework (by shortening treatment initiation by 1.2 days on average) could reduce diagnostic delay-associated health costs by EUR 2.8 billion/year in the EU. Using a productivity-loss coefficient of EUR 160/day, an economic benefit of ca. EUR 192/patient has been calculated [86].

Clinical trials, albeit few, have demonstrated the societal impact of POCT in improving primary health in the general population, in particular, by improving control of diabetes, lipidemia and respiratory infections, as shown in the following Table 4.

## 5. Pharmacist Training and Education for POCT Services

The provision of POCT services in pharmacies necessitates adequate training and education for pharmacists. Both pre-service and in-service training programs are essential for equipping pharmacists with the necessary skills and competencies to effectively deliver point-of-care services. Training programs should cover a range of topics, including clinical knowledge, point-of-care testing procedures, quality control, data interpretation, communication skills and patient management [89]. Pre-service education should be integrated into pharmacy curricula to ensure that future pharmacists are adequately prepared to provide point-of-care services.

The introduction of analytical measurement into community pharmacies represents not only a regulatory expansion of professional scope but also a profound shift in the epistemology of pharmaceutical science: the pharmacist must acquire analytical literacy comparable to that of a laboratory technologist [90].

Unlike traditional compounding or dispensing activities, POC testing requires quantitative reasoning, calibration control and understanding of chemical interferences—skills typically associated with analytical chemistry curricula.

Undergraduate pharmacy programs increasingly integrate *applied analytical chemistry* modules that cover the principles of spectrophotometry, electrochemistry and immunoassay kinetics. However, global curricula audits revealed that the majority (>50%) of the surveyed curricula lacked formal routine review for laboratory medicine [91].

A competency-based model for diagnostic education in pharmacy should include

1. *Analytical Chemistry Core*—stoichiometry of assays, Beer–Lambert law, electrochemical potentials and reaction equilibria.
2. *Instrumental Analysis*—calibration curves, signal processing, blank subtraction and sensitivity calculation.
3. *Bioanalytical Techniques*—enzyme kinetics, antibody–antigen binding thermodynamics (Ka, Kd) and enzyme-linked detection.
4. *Quality Management*—statistical process control, uncertainty estimation and ISO 15189 principles.
5. *Clinical Correlation*—translation of numerical results to therapeutic decisions.

Educational institutions should collaborate with relevant stakeholders to develop standardized training programs and guidelines for the integration of point-of-care services into pharmacy education. Continuing education programs should be developed and implemented to provide ongoing training and support for practicing pharmacists. These programs can be delivered through various formats, including workshops, seminars, online courses and on-the-job training. Collaboration between pharmacy organizations and educational institutions is crucial for the successful implementation of continuing education programs. Support and resources should be provided to facilitate the implementation of training and education programs [92]. This includes the development of training materials, guidelines and resources for pharmacists and educational institutions. Financial and logistical support may also be necessary, particularly for low-resource settings. Collaboration and partnerships among stakeholders are vital for the successful training and education of pharmacists in point-of-care services. By working together, stakeholders can share resources, knowledge and expertise, ensuring that pharmacists are adequately trained and supported in their delivery of point-of-care services.

## 6. Integration of Digital Technologies, Mobile Health (mHealth) and Artificial Intelligence into Point-of-Care Services

### 6.1. Integration with Telepharmacy and Remote Supervision

Advancements in technology have facilitated the development and utilization of innovative tools that enhance the efficiency and effectiveness of point-of-care services. These technologies can be broadly categorized into telemedicine, mobile health applications and health information systems, which provide unique advantages to pharmacies and their clientele. Telemedicine allows for real-time remote consultations between healthcare professionals and patients, resulting in time, cost and convenience savings, especially for those living in rural areas [93,94]. Telepharmacy, a unique aspect of telemedicine, enables the provision of pharmaceutical care through digital means, allowing pharmacists to perform medication reviews, provide counseling and confirmation of prescriptions and assess the appropriateness of therapy, dosage and potential drug interactions. Telepharmacy has improved medication adherence rates and the overall health of patients in several states and various countries [95].

Health information systems, comprising equipment and processes that collect, manage and analyze healthcare data, provide valuable benefits such as automation that enhances efficiency and control over data management, improved operational performance and the ability to aggregate information and analyze it statistically to improve the quality of services [96,97]. These systematic and automatic approaches to managing health information help minimize errors associated with manual handling and provide abundant and quick access to telecommunication possibilities. These widely used systems merit further exploration.

Despite the evident social benefits of POCT services, their uptake and development among pharmacies remain inadequate. Barriers such as insufficient knowledge of the services, concerns regarding the impact on patient health and pharmacy workload and doubts about the viability and profitability of point-of-care services impede the widespread implementation of these services [70]. Young and newly graduated pharmacists perceive greater potential for expanding these services compared to older pharmacists nearing retirement, suggesting a viewpoint influenced by their professional experience. While it is considered vital to actively promote the development of point-of-care services, attempts to increase awareness, participation or professional capacity must be tailored to the unique characteristics of target populations [98].

### 6.2. Artificial Intelligence and Decision Support for Pharmacy-Based Point-of-Care Testing

The increasing digitization of diagnostic workflows in community pharmacies creates fertile ground for the integration of artificial intelligence (AI) and advanced chemometric decision-support systems [99]. While contemporary point-of-care (POC) devices already incorporate embedded algorithms for calibration, baseline correction and basic signal interpretation, AI extends this analytic layer toward multivariate pattern recognition, risk stratification and individualized therapeutic recommendations. In pharmacy-based POCT, AI may operate at three conceptual levels: (i) signal-level processing, (ii) result-level interpretation and (iii) population-level learning and feedback [100]. However, the introduction of AI at each of these levels raises important methodological, regulatory and epistemological challenges that remain incompletely resolved.

At the signal-processing level, machine-learning (ML) models are increasingly used to enhance interpretation of raw analytical signals generated by electrochemical, optical or immunochemical sensors [101]. Traditional calibration approaches based on linear or polynomial regression are often insufficient in real-world pharmacy environments, where non-linear sensor behavior, matrix effects, environmental variability and time-dependent drift are common. ML approaches—such as support-vector regression (SVR), random forests, artificial neural networks (ANNs) and convolutional neural networks (CNNs)—can learn complex, non-linear mappings between raw sensor outputs and reference laboratory values, correcting implicitly for temperature fluctuations, lot-to-lot reagent variability and interfering species [102].

Nevertheless, this apparent robustness introduces a critical controversy: model opacity and overfitting risk. Many ML-based calibration models function as “black boxes,” offering limited interpretability regarding how specific corrections are derived [103]. In regulated diagnostic settings, this lack of transparency challenges traceability, metrological accountability and compliance with standards such as ISO 15189, ISO 22870 and the EU IVDR. Moreover, models trained on large but context-specific datasets may perform well during validation yet degrade when deployed in pharmacies with different patient populations, workflows or environmental conditions—a phenomenon known as dataset shift. The absence of standardized protocols for continuous post-deployment performance auditing of AI-calibrated POCT systems remains a significant unresolved issue.

These challenges become even more pronounced in multiplex POC platforms, where multiple analytes are measured simultaneously and overlapping spectral or electrochemical signatures occur. Although multivariate calibration techniques (e.g., partial least squares or principal component regression) combined with non-linear ML models can deconvolute overlapping responses and maintain low bias and coefficient of variation (CV), they also amplify dependency on high-quality, representative training datasets. Small biases at the signal level may propagate non-linearly across analytes, potentially leading to clinically relevant misclassification—particularly when results approach clinical decision thresholds.

At the result-interpretation level, AI is increasingly deployed as a clinical decision-support system (CDSS) embedded in pharmacy workflows. Once a quantitative POC value (e.g., HbA1c, CRP, lipid profile or troponin) is generated, it must be contextualized using patient-specific variables such as age, comorbidities, medication history and prior results [104]. Rule-based systems derived from clinical guidelines offer transparency and regulatory familiarity, but they lack flexibility in handling complex, borderline or atypical cases. More advanced AI approaches—such as gradient-boosted decision trees or Bayesian networks—can integrate multiple biomarkers and longitudinal data to generate individualized risk scores [105].

This AI-assisted interpretation is particularly relevant in community settings where pharmacists may have limited time and incomplete access to full medical records [106]. By embedding evidence-based algorithms—derived from clinical-trial and real-world outcome data—into the pharmacy information system, the POC result can immediately trigger suggested actions: intensification of lifestyle counseling, recommendation to consult a physician, medication review or repeat testing after a defined interval.

However, these approaches raise concerns regarding clinical accountability and cognitive bias. AI-generated outputs, even when presented as traffic-light indicators or probabilistic risk estimates, may exert disproportionate influence on pharmacist decision-making, particularly in high-throughput or time-constrained settings. This “automation bias” risks shifting clinical judgment from human professionals to algorithmic recommendations whose training data, assumptions and failure modes may not be fully understood by end users. Importantly, responsibility for adverse outcomes remains legally and ethically assigned to healthcare professionals, not algorithms—yet current regulatory frameworks provide limited guidance on how AI-assisted decisions should be documented, audited or contested.

Furthermore, at the population-learning level, AI systems aggregating pharmacy POCT data offer powerful opportunities for epidemiological surveillance, quality control and adaptive model improvement. Nonetheless, this layer introduces unresolved challenges related to data governance, privacy, bias amplification and feedback loops. If AI models are continuously retrained on real-world data without rigorous external validation, systematic errors or local practice biases may be reinforced rather than corrected. Federated learning and privacy-preserving analytics offer partial solutions, but their implementation in community pharmacy networks remains technically complex and unevenly regulated.

In summary, while AI-enhanced POCT in pharmacies holds substantial promise for improving analytical robustness and clinical relevance, its widespread adoption is constrained by unresolved questions surrounding model transparency, generalizability, regulatory acceptance and professional accountability. Addressing these challenges will require not only technical advances but also the development of standardized validation frameworks, continuous performance monitoring schemes and clear delineation of human–AI responsibility in decentralized diagnostic care.

The impact of AI in pharmacy-based point-of-care testing can be assessed using a combination of pre–post and controlled-comparison metrics spanning analytical, clinical, operational and system-level domains. At the analytical level, improvements in accuracy, precision, drift correction and test validity before and after AI-assisted calibration—or relative to non-AI workflows—provide objective evidence of added value. Clinically, AI impact can be measured through changes in decision concordance with guidelines, referral rates and inter-operator variability. Operational metrics such as time to result, staff time per test and throughput capture workflow efficiency, while patient-reported satisfaction and trust reflect service quality. Finally, system-level and governance metrics—including prescribing patterns, model stability, override frequency and explainability indicators—are essential to demonstrate not only performance gains but also the reliability, accountability and sustainability of AI-assisted POCT in real-world pharmacy settings.

**Figure 6:**
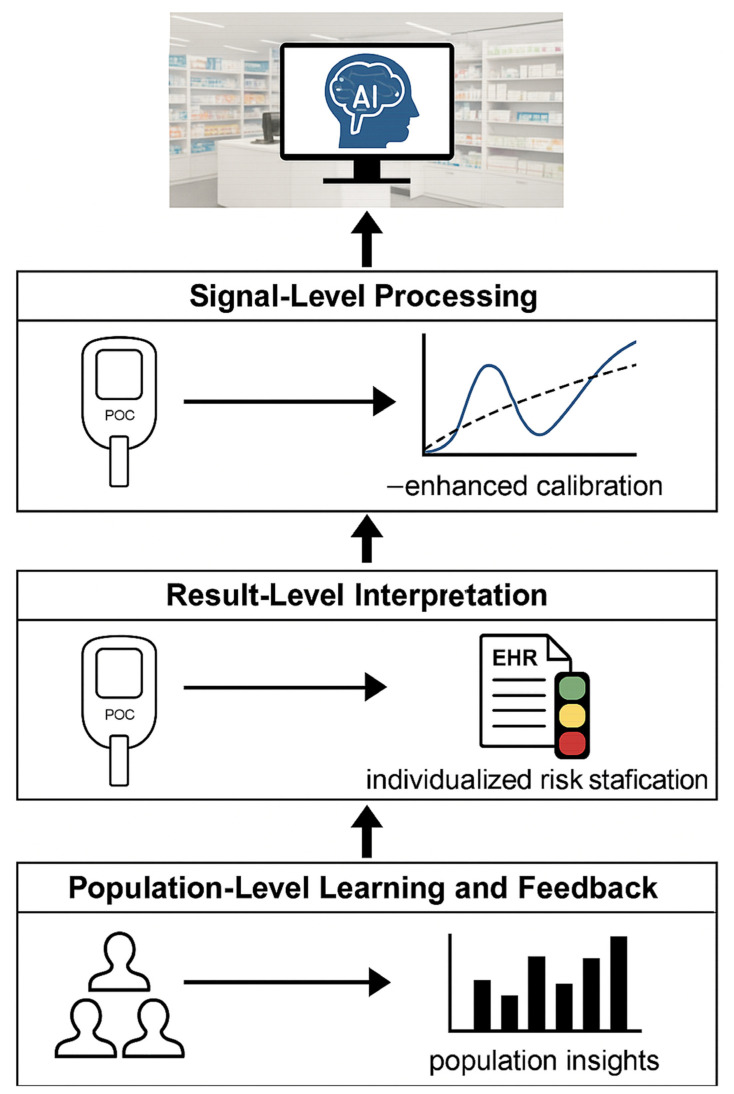
Schematic representation of AI integration across the three analytical and clinical layers of pharmacy-based point-of-care testing (POCT). At the signal-processing level, machine-learning models enhance raw electrochemical, optical and immunochemical signals by correcting for drift, matrix effects and non-linearities. At the result-interpretation level, AI-driven clinical decision-support systems combine biomarker values (e.g., HbA1c, CRP, lipid profile) with patient-specific factors and electronic health records to generate individualized risk estimates. At the population-learning level, aggregated POCT data contribute to network-wide surveillance, anomaly detection and continuous model improvement, transforming pharmacies into nodes of a learning diagnostic ecosystem.

The different levels of AI integration across the three analytical and clinical layers of pharmacy-based POCT are graphically summarized in the following Figure 6.

### 6.3. Population-Level Learning and Continuous Performance Optimization

Telepharmacy and mHealth applications further enhance communication, enabling remote consultations and electronic record integration. AI and machine learning support decision-making through automated result interpretation, risk stratification and clinical recommendations. Health-information systems interlink data from pharmacy tests with national surveillance networks, improving epidemic response and pharmacovigilance. At the population level, pharmacy POC networks generate large volumes of high-frequency analytical data [107]. AI-based analytics applied at this scale can detect trends that are invisible at the individual-pharmacy level, such as emerging outbreaks, systematic device drift or geographically clustered anomalies [108].

Unsupervised learning methods (e.g., clustering, anomaly detection) can identify pharmacies whose QC performance deviates from the peer distribution, prompting targeted technical support or recalibration. Similarly, time-series models applied to anonymized POC markers (e.g., CRP, influenza antigen tests) across regions can serve as early-warning systems for respiratory epidemics or other public-health threats [109]. Crucially, these AI models can be continuously updated through federated-learning frameworks, where model training occurs locally at each node (pharmacy) and only model parameters, not raw patient data, are shared [110]. This approach enhances privacy while still allowing global improvement of predictive performance.

## 7. Future Directions, Implementation Barriers and Opportunities for Pharmacies in Point-of-Care Services

The future of pharmacies in point-of-care services appears promising amidst a dynamic and evolving healthcare landscape. The COVID-19 pandemic has underscored the importance of pharmacies in public health response and community-based care. This pivotal role creates new opportunities for pharmacies to expand and enhance healthcare services, particularly in testing and monitoring. With growing consumer demand for healthcare services that are convenient, efficient and accessible, the use of portable testing devices or kits for screening and monitoring health and disease is increasing. To take advantage of this growth, there are significant opportunities for pharmacies to establish themselves as competent entrepreneurs in their communities, particularly in chronic disease management, wellness and preventive care and infectious disease screening.

With digital technologies advancing rapidly and poised to reconfigure the pharmacy landscape, a revolutionary transformation of pharmacy services is becoming more probable, facilitated by emerging technologies such as AI, big data analytics, mHealth and digital health. There is an opportunity for pharmacies to embrace these emerging technologies to redesign traditional pharmacy services to become more value-added. Big data analytics can help analyze health data at the population level to generate insights to improve wellness and preventive healthcare services. Digital health and affiliated technologies can help automate the drug dispensing process and the flow of drug-related information, thereby improving the efficiency and effectiveness of pharmacy services and freeing up pharmacists to perform healthcare services.

Despite the many opportunities in point-of-care services, there are several potential challenges that may inhibit the growth and sustainability of these services in pharmacies. These potential challenges include deficiencies in pharmacy management and operation, pharmacy and pharmacist reimbursement, pharmacy and pharmacist education and training and pharmacy-facilitated patient education and information. Because these services are pharmacy-based, management and operation are crucial to the success of these services [111]. There is a lack of management policies, strategies and guidelines to help pharmacy managers run and administer pharmacy-based services effectively and efficiently [112]. This deficiency may become a stumbling block to the initiation and operation of pharmacy-based services. Lack of reimbursement for pharmacy-based services is one of the main barriers to the growth and sustainability of these services worldwide, along with knowledge gaps regarding device accuracy and maintenance and the resistance from other healthcare providers unaware of pharmacists’ diagnostic competencies [113]. In this context, organized interprofessional collaboration between pharmacies and other healthcare providers is scarce. Non-collaborative pharmacy practices are a barrier to pharmacists’ participation in established collaborative roles. Lack of knowledge regarding other providers’ collaborative roles is a barrier for healthcare providers, especially physicians, to refer patients to collaborative pharmacy services. Thus, raising awareness is vital to implement potential interprofessional collaboration practices. Finally, professional associations could play a crucial role in establishing accreditation and continuous professional-development pathways.

Patient perspectives and satisfaction with pharmacy point-of-care services are equally important factors that can affect the effectiveness of these services. Such would, for example, include administrative efficiency (waiting area, opening hours, etc.), technical competency (professionalism of the personnel, technical expertise) and convenience of the location. Relevant studies have indicated that administrative and technical competency, including pharmacist attitude, were the most important factors influencing prospective patients to opt out of pharmacy-based POCT services [114,115]. Understanding patient perspectives and factors that predict satisfaction is necessary to ensure the continuous provision of these services by community pharmacies in the future [116]. Previous research indicated that offering an easy definition and providing examples of services can enhance patient understanding. Respondents expressed a strong desire for more decentralized health services, especially those covered by the health insurance system [117]. However, desired services providing free treatments caused a negative reaction. While patients wanting more services tended to be older and have a higher number of prescriptions, these characteristics could not be directly linked to desired pharmacy point-of-care services. Services classified as important did not correlate with a high percentage of respondents desiring the service [118].

The core pharmacist competencies and challenges for realizing pharmacy-based POCT services are summarized in the following Table 5, while region-wise reimbursement and policy frameworks are summarized in Table 6:

Despite the perceived clinical and economic benefits of pharmacy-based POCT, its large-scale implementation could also face substantial practical constraints related to infrastructure, staffing and workflow integration. One of the most frequently cited barriers is space and privacy. Surveys across Europe and North America suggest that only part of community pharmacies currently meet recommended requirements for a dedicated consultation or testing area that ensures visual and acoustic privacy, biosafety and workflow separation from dispensing activities, for example, when implementing HIV POCT [123]. Smaller, high-throughput urban pharmacies are disproportionately affected, often lacking sufficient floor area to accommodate POCT without structural redesign.

Workflow disruption and limited human resources present additional practical challenges. POCT introduces pre-analytical (sample collection, patient consent), analytical (test execution, QC) and post-analytical (result interpretation, documentation, referral) steps that are not natively aligned with traditional pharmacy workflows [124]. Without dedicated scheduling or appointment systems, ad hoc testing can create bottlenecks, increase waiting times and elevate the risk of procedural errors, especially during peak dispensing hours [123].

The following Table 7 summarizes recommendations that directly reflect findings on regulatory heterogeneity, workforce preparedness, quality assurance gaps, economic sustainability and the emerging challenges of AI-assisted diagnostics in community pharmacies. Collectively, they aim to support safe scaling, professional credibility and long-term integration of pharmacies into decentralized, digitally enabled healthcare systems.

## 8. Conclusions

The expanded role of the pharmacy workforce, including pharmacists as direct care providers, is a growing trend throughout the world. Pharmacy-based point-of-care services redefine the pharmacist’s role as a clinical decision-support professional. They can deliver faster diagnostics, personalized interventions and improved public-health outcomes.

Technological progress and favorable policy will continue to expand this domain. The challenge for the coming decade is ensuring consistent education, equitable reimbursement and digital interoperability, transforming every community pharmacy into a connected health hub. The Global Strategy on Human Resources for Health includes the expansion of practice roles and responsibilities, in conjunction with an increase in the number of students entering pharmacy education programs and the establishment of pharmacy schools, as key global workforce strategies addressing medication-related health problems [125]. Pharmacists will need to implement direct patient care services, including but not limited to medication therapy management, immunizations, smoking cessation counseling, disease management, health screenings and other patient care services. The ability to provide expanded patient care services will depend on the practice environment and workplace setting, regulations and laws governing pharmacists’ scope of practice and reimbursement policies governing payments for pharmacy services.

## Figures and Tables

**Table 1:** Main categories and target applications of CLA-waived POCT available in community pharmacies.

Test Category	Target Application	Reference
Lateral flow immunoassay	Group A Streptococcus Influenza A and B COVID-19 Human immunodeficiency virus (HIV) Hepatitis C virus (HCV) Helicobacter pylori Mononucleosis Syphilis	[24] [25] [26] [27] [28] [29] [30] [31]
Molecular-based	Chlamydia Gonorrhea Trichomonas Group A Streptococcus Influenza A and B COVID-19 Respiratory syncytial virus (RSV)	[32] [32] [32] [33] [34] [35] [36]

**Table 2:** Anticipated key benefits and challenges of pharmacy-based POC services.

Domain	Benefits	Challenges
Healthcare system	Reduced hospital burden; faster diagnosis	Variable reimbursement; policy gaps
Pharmacy sector	Diversified revenue; enhanced visibility	Infrastructure investment
Patients	Convenience; improved adherence *	Perception of test reliability
Society	Better disease control; antimicrobial stewardship	Need for regulation and data protection

* Reduction of antibiotics [70,71], a lifestyle-based glycemic control [72].

**Table 3:** Cost breakdown for adopting a POCT service at the pharmacy (estimates).

Cost Category	Cost (in EUR)
Equipment (read-out device)	2000–6000
Quality control	300/year
Test reagents	1–2/test
Software license and update, IoT connectivity	200/year

**Table 4:** Representative clinical trials assessing the impact of validated pharmacy-compatible POCT systems on primary health improvement.

Analyte	Device Type	Analytical R^2^ vs. Laboratory	Clinical Outcome Improvement	Reference
HbA1c	Optical photometry	0.97–0.99	Same-visit HbA1c testing improved diabetes control; ~7–10% more patients reached target HbA1c	[87]
Total cholesterol	Electrochemical	0.95–0.98	Immediate lipid results increased statin treatment adherence by ~10–15%	[88]
CRP	Lateral-flow immunoassay	0.93–0.97	Reduced unnecessary antibiotic prescribing in respiratory infections	[71]

**Table 5:** Core pharmacist competencies and implementation barriers for POC services.

Category	Key Competencies	Common Barriers
Clinical interpretation	Result analysis, referral decisions	Lack of training, time constraints
Technical skills	Device operation, QC procedures	Cost of equipment, maintenance
Communication	Counseling, confidentiality	Space limitations, privacy issues
Management	Record keeping, workflow integration	Inconsistent reimbursement

**Table 6:** Summary of reimbursement and policy frameworks by region.

Region	Reimbursement Mechanism	Policy Instruments	References
European Union	National health service contracts	Standardized accreditation, IVDR 2017/746	[113]
North America	Insurance & Medicare billing codes	CLIA-waived classification; collaborative practice	[119]
Asia-Pacific	Mixed private/public schemes	Pharmacy-led screening programs, national POCT standards	[120]
Africa	NGO and donor support	Capacity-building projects, malaria/HIV POCT policies	[121,122]

**Table 7:** Actionable recommendations for key stakeholders in pharmacy-based POCT.

Stakeholder	Priority Area	Actionable Recommendation
Policymakers and Regulators	Regulatory clarity	Establish explicit national frameworks recognizing community pharmacies as decentralized diagnostic units, aligned with CLIA-waived principles (USA) or IVDR 2017/746 (EU).
	Quality and safety	Mandate participation of pharmacies in standardized internal quality control (IQC) and external quality assessment (EQA) schemes, proportionate to test complexity.
	AI governance	Introduce regulatory guidance for AI-assisted POCT covering transparency, post-market performance monitoring, dataset shift management and accountability under medical-device legislation.
	Reimbursement	Develop sustainable reimbursement models that value analytical accuracy and reduced diagnostic delay (e.g., value-of-information–based reimbursement rather than fee-per-test alone).
Educational Institutions and Professional Bodies	Curriculum design	Integrate applied analytical chemistry, biosensor principles, quality management (ISO 15189:2022 and 22870:2016) and diagnostic interpretation into undergraduate pharmacy curricula.
	Continuing education	Implement accredited, competency-based continuing professional development programs focused on POCT operation, quality control, data interpretation and AI-assisted decision support.
	Interdisciplinary training	Promote joint training modules involving pharmacists, laboratory professionals and clinicians to strengthen interprofessional trust and referral pathways.
Pharmacy Owners and Managers	Infrastructure and workflow	Invest in dedicated POCT spaces ensuring biosafety, privacy and controlled environmental conditions, with clear workflow separation between testing and dispensing activities.
	Quality management	Establish simplified but robust quality management systems (QMSs) including documentation, traceability, routine QC review and corrective-action protocols.
	Strategic positioning	Position POCT as a value-added professional service integrated with counseling, referral and preventive care, rather than as a standalone retail offering.

## Data Availability

No new data were created or analyzed in this study.

## Abbreviations

The following abbreviations are used in this manuscript:

AI	Artificial Intelligence
ANNs	Artificial Neural Networks
Au NPs	Gold (Au) Nanoparticles
CLIA	Clinical Laboratory Improvement Amendments
CMS	Centers For Medicare & Medicaid Services
CLIA	Chemiluminescent Immunoassays
CDSS	Clinical Decision-Support System
CV %	Coefficient Of Variation
CNNs	Convolutional Neural Networks
CPT	Cost-Per-Test
CPAR	Cost-Per-Accurate-Result
CRP	C-Reactive Protein
EHR	Electronic Health Record
EQA	External Quality Assessment
GDPR	General Data Protection Regulation
Hba1c	Glycated Hemoglobin
HIPAA	Health Insurance Portability and Accountability Act
HIV	Human Immunodeficiency Virus
HCV	Hepatitis C Virus
IQC	Internal Quality Control
IVDR	In Vitro Diagnostic Regulation
JCTLM	Joint Committee For Traceability In Laboratory Medicine
LAMP	Loop-mediated isothermal amplification
LOD	Limit Of Detection
LOQ	Limit Of Quantification
ML	Machine Learning
μTAS	Micro-Total-Analysis Systems
mHealth	Mobile Health
NAB	Net Analytical Benefit
NAATs	Nucleic Acid Amplification Technologies
PLS	Partial-Least-Squares Regression
POC	Point-Of-Care
POCT	Point-Of-Care Testing
PCA	Principal Component Analysis
QC	Quality Control
QMS	Quality Management System
RPA	Recombinase Polymerase Amplification
RSV	Respiratory Syncytial Virus
SVR	Support-Vector Regression
TGA	Therapeutic Goods Administration
VOI	Value-Of-Information
